# Image-guided Pro-angiogenic Therapy in Diabetic Stroke Mouse Models Using a Multi-modal Nanoprobe: Erratum

**DOI:** 10.7150/thno.114285

**Published:** 2025-04-19

**Authors:** Ying-Ying Bai, Xihui Gao, Yuan-Cheng Wang, Xin-Gui Peng, Di Chang, Shuyan Zheng, Cong Li, Shenghong Ju

**Affiliations:** 1 Jiangsu Key Laboratory of Molecular and Functional Imaging, Department of Radiology, Zhongda Hospital, Medical School, Southeast University, Nanjing, 210009, China;; 2 Key Laboratory of Smart Drug Delivery, Ministry of Education & PLA, School of Pharmacy, Fudan University, Shanghai, 201203, China.

The authors apologize that some incorrect representative images were accidentally used in our previously published paper when the first author assembled the figures, including the MR images in Figure 3A, the *in vivo* NIRF images in Figure 3C, and the immunohistochemical image in Figure 6A. The corrected figures are shown below. The authors declare that these corrections do not change the results or conclusions of their paper. The authors sincerely apologize to the Journal and its readers for the confusion this may have caused.

## Figures and Tables

**Figure 3:**
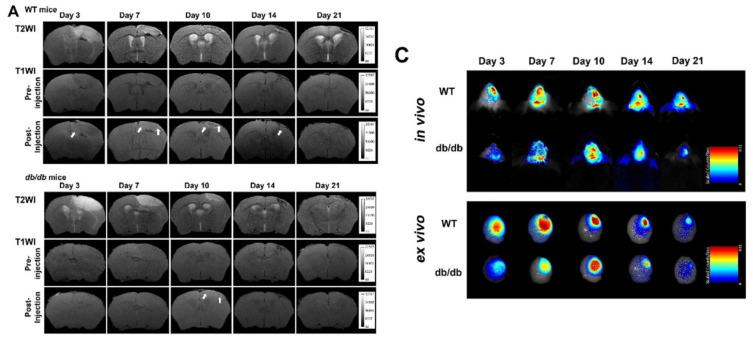
Corrected figure for original Figure 3A and C.

**Figure 6:**
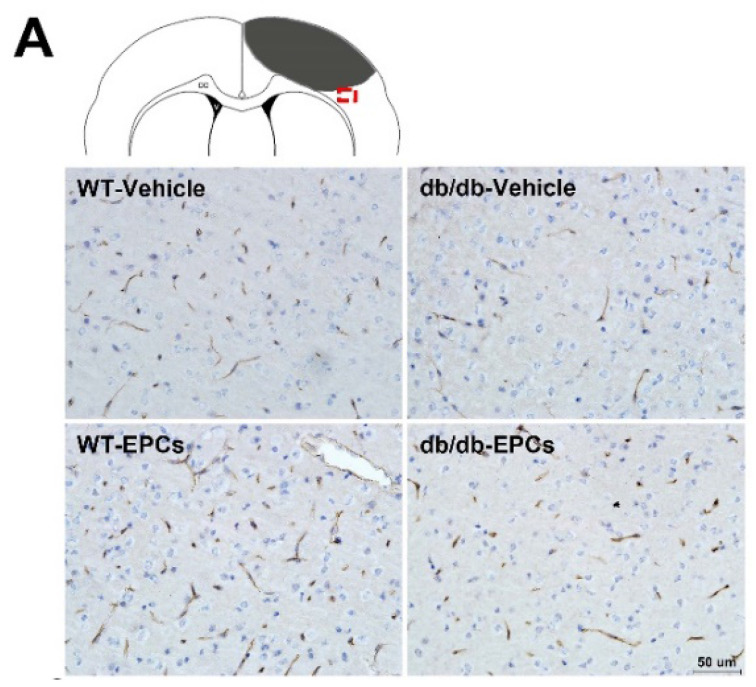
Corrected figure for original Figure 6A.

